# Innovative Surgical Management of Nasal Septal Lipoma: A Report of a Rare Case and Review of Literature

**DOI:** 10.7759/cureus.89724

**Published:** 2025-08-10

**Authors:** Al Thuraya Al Sinani, Asma Al Junaibi, Janan Al Abduwani, Maimuna Al-Saadi, Salwa Habib, Ahmed Al Shaqsi, AbdulAziz Al Azri

**Affiliations:** 1 Otolaryngology - Head and Neck Surgery, Al Nahdha Hospital, Muscat, OMN; 2 Pathology, Khoula Hospital, Muscat, OMN; 3 Radiology, Al Nahdha Hospital, Muscat, OMN

**Keywords:** composite graft, lipoma, nasal obstruction, nasal septum, septal mass

## Abstract

Nasal septal lipoma is a very rare entity that is encountered in otolaryngology, head and neck surgery, with only 16 reported cases in the literature, to the best of our knowledge. We have reported a child with an isolated septal lipoma without any associated syndromes or intracranial extension.

Surgical excision was done, and the lipoma was found to be adherent to the nasal mucosa, so complete excision of the mass with its nasal mucosa was performed, and reconstruction of the defect was done using a cartilage-skin composite graft of the involved area. To our knowledge, this is the first case to be reconstructed using this technique and with complete healing of the nasal mucosa. A comprehensive review of the reported cases is done in this case report.

## Introduction

Lipoma is one of the most common benign tumors with a prevalence of 1% in the general population [1,2]. It represents around 13% of the head and neck region [1,3]. It is very rare in patients under the age of 20 years, with peak incidence between the fourth and sixth decades [2]. However, lipomas are extremely rare to be found in the nasal cavity and paranasal sinuses, with very few reported cases in the literature [4]. Lipomas in the nasal tip, vestibule, and alar crease have been reported as well, but to a lesser extent [5]. Here we report a case of septal lipoma in pediatric patients, highlighting the surgical management technique. 

## Case presentation

**Figure 1:**
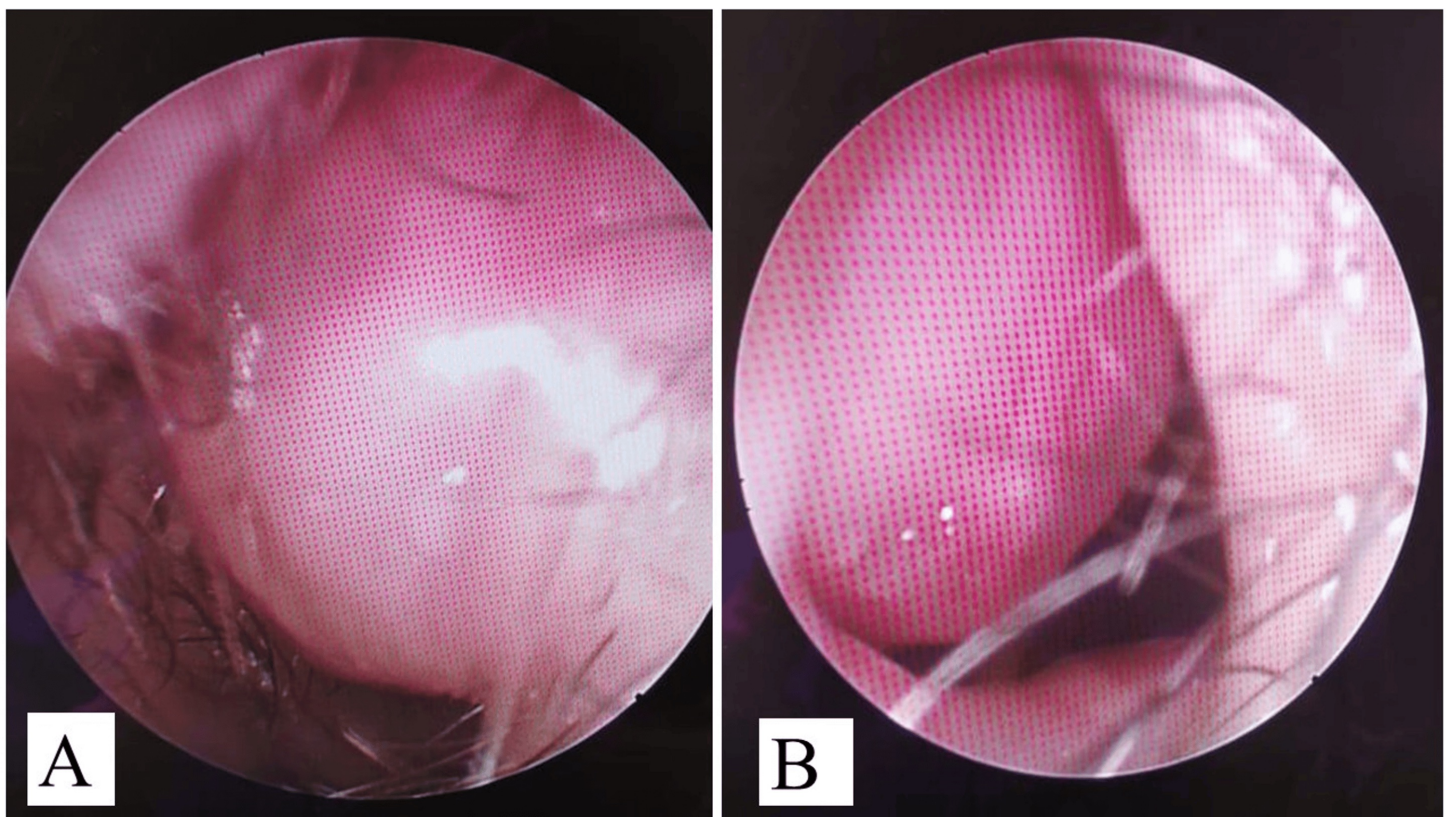
Endoscopic findings that show (A) right nasal cavity with deviated nasal septum, and (B) smooth septal bulge in the left nasal cavity that was soft to palpation (lipoma)

A seven-year-old girl presented with bilateral nasal obstruction for a 10-month duration with a progressively growing intranasal swelling that was noticed by her parents. It was associated with snoring and aching type headaches, but no history of epistaxis, nasal pain, or any other nasal symptoms. Her past medical, surgical, and family history was unremarkable. Examination, including rigid nasal endoscopy, showed right hard septal swelling (deviated nasal septum) and left soft superior septal swelling with no extension to the nasopharynx or oropharynx. The swelling was around 2x2 cm in diameter, not tender, non-compressible, and it was not bleeding on touch, as shown in Figure 1.

**Figure 2:**
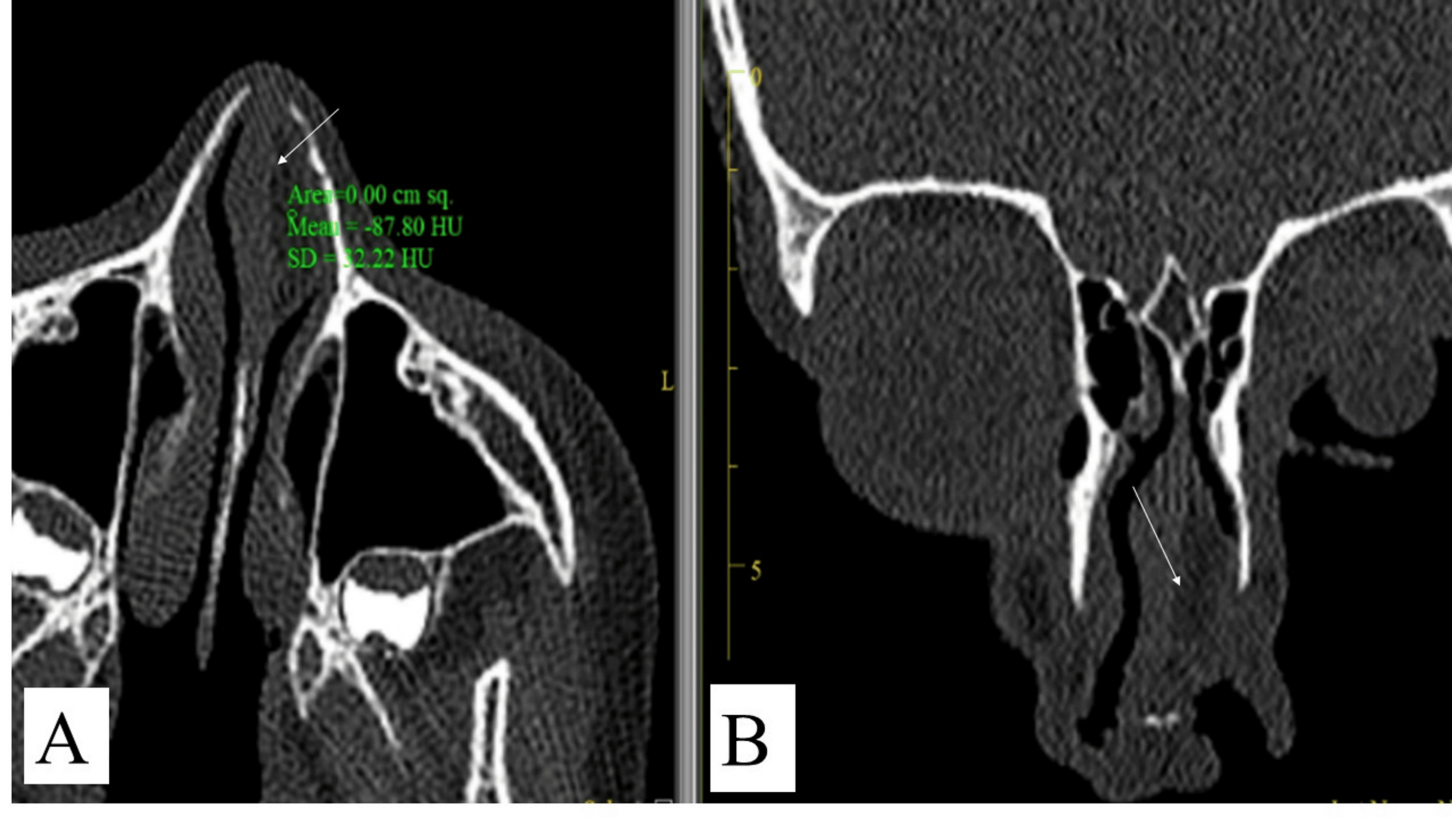
CT of nasal septal lipoma A) axial section; B) coronal view showing left anterior septal fatty lesion with mean Hounsfield unit (HU) – 88, homogenously hypodense representing lipomatous lesion

**Figure 3:**
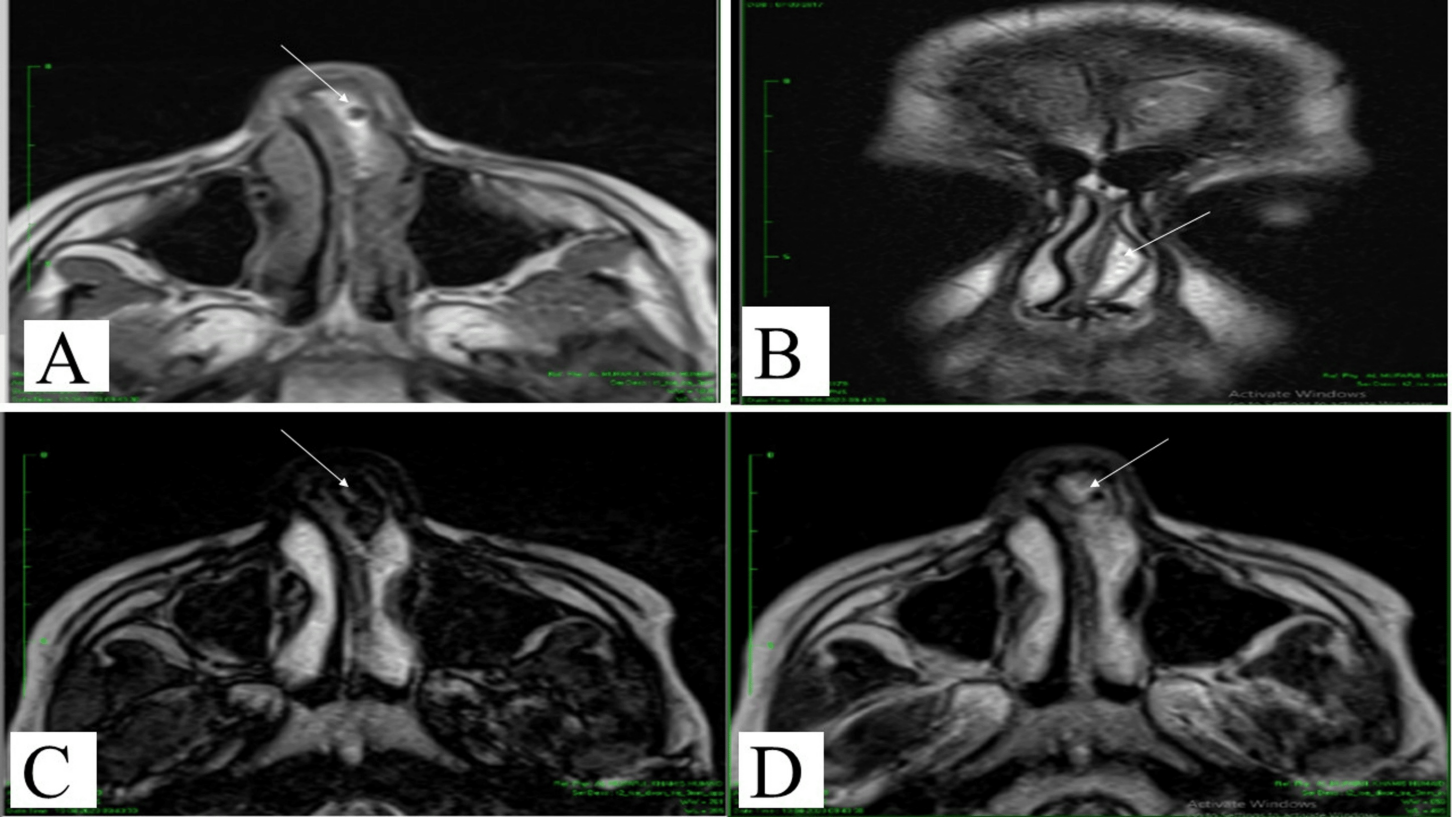
MRI of the septal lipoma A) Axial T1 MRI image showing homogenous high signal intensity at the left side of the nasal septum. B) Coronal T2 MRI image showing intermediate high signal intensity at the left side of the nasal septum. C, D) Dixon MRI (C out, D in) sequences. C) shows low signal intensity at the site of the lesion, representing fat suppression. D) shows high signal intensity at the site of the lesion, representing fat. MRI features are consistent with lipoma.

Computed tomography (CT) scan of paranasal sinuses was done, and it showed a left anterior septum hypodense lesion with a deviated nasal septum to the right. Magnetic resonance imaging (MRI) was done as well, and it showed an ovoid lesion in the left anterior septum that measures 20x8x19 mm in dimensions, and it demonstrated high T1 and T2 signals with low signals in saturation sequences. These features were consistent with lipoma. There were no intracranial extensions or other lesions seen as demonstrated in Figure 2 and Figure 3.

**Figure 4:**
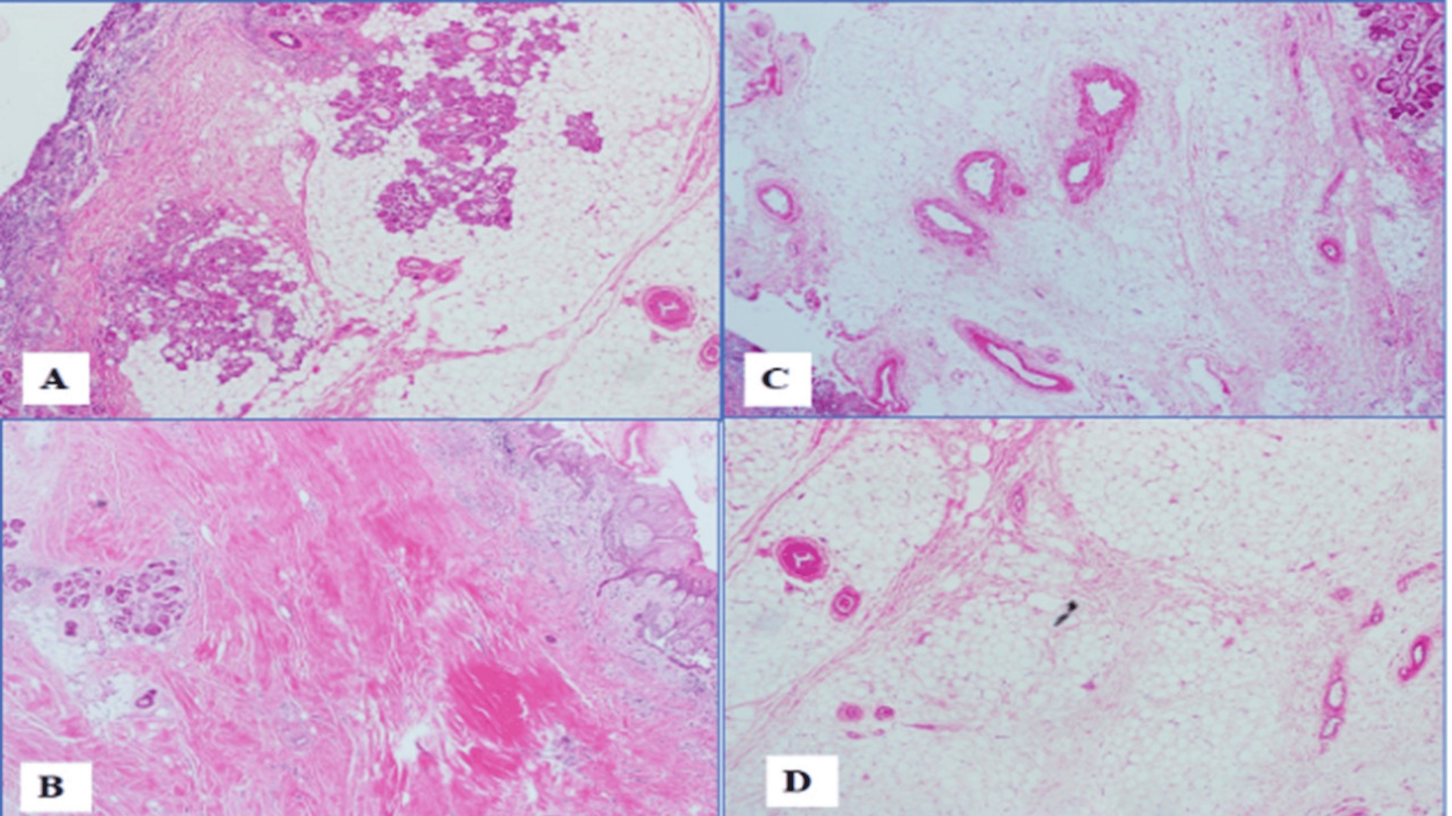
Histopathology (A) Low power view (H&E, 2x) showing ulcerated surface (top left corner), with underlying minor salivary glands with prominent adipose tissue and thick-walled blood vessels (in C, D, 4x). (B) Low power view (H&E, 2x) showing squamous mucosa (top right corner) with underlying fibrosis and scarring.

**Figure 5:**
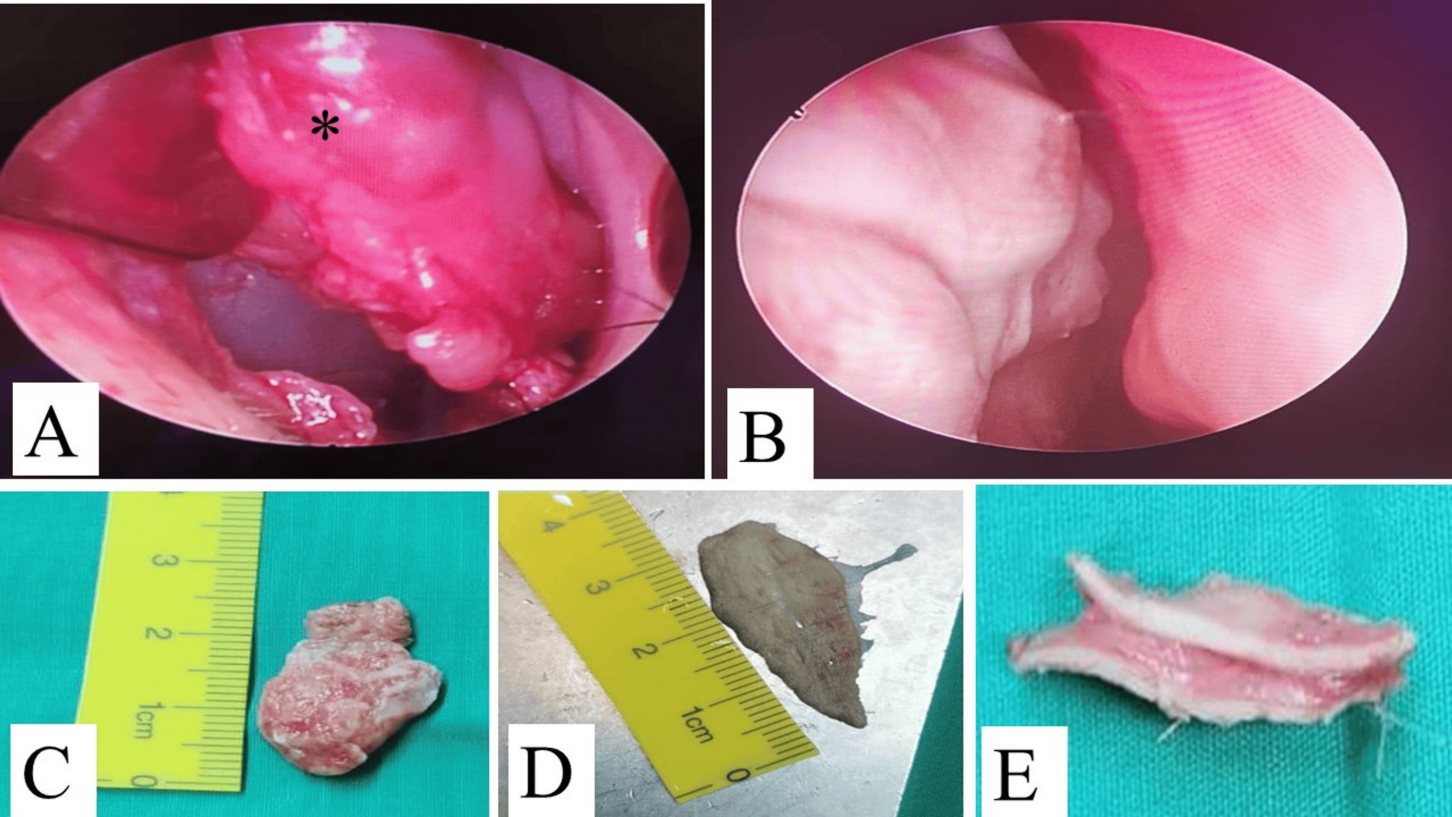
Intraoperative findings A) Mucoperichondrium flap elevated with the lipoma from the septal cartilage (astrix) that was intact, but the lipoma was very adherent to the flap. B) endoscopic examination after four weeks post-surgery and removal of the nasal splint, well-healed nasal mucosa. C) intra op excised lesion with the nasal mucosa, measures approximately 25 mm. D) left conchal composite graft with cartilage and skin, skin measures 30 mm as shown in E.

The patient underwent surgical excision of the lipoma with endoscopic monitoring under general anesthesia. Intraoperatively, the lesion was found to be separable from the septal cartilage but very adherent to the mucoperichondrial flap, as shown in Figure 4. The mass was excised in toto along with the adherent nasal septal mucosal flap and around 1 cm of the caudal septum, as it was thin and weak. Reconstruction was done using a composite graft that was harvested from the left ear (conchal cartilage and skin) via post-auricular excision (1x1 cm cartilage island and 3x1.5 skin). The cartilage was used as a caudal septal replacement graft to prevent future saddle nose deformity. The full-thickness skin was used for reconstruction of the nasal mucosal defect. Bilateral intranasal sialastic splints were kept for four weeks to ensure graft uptake. Histopathological examination confirmed the diagnosis of lipoma (Figure 4). Four weeks postoperatively, the patient was taken for the removal of the nasal splints and an examination under general anesthesia, which revealed well-healed nasal mucosa and excellent uptake of the graft as shown in Figure 5. No recurrence of symptoms or lesion was noted until 6 months post-surgery.

## Discussion

Lipoma is a common, slow-growing benign neoplasm that is most commonly found in the trunk, neck, upper and lower extremities [3]. It is slightly more predominant in males [1,2]. In the head and neck area, lipomas are seen in the posterior neck, cheeks, tongue, floor of the mouth, and buccal sulcus, which are the most common locations [1]. Facial lipomas are very rare and account for 1.6% of all facial masses [3,6]. Lipomas can occur subcutaneously or submucosally, and due to the paucity of adipose tissue in the nasal cavity and paranasal sinuses, lipoma is very rarely seen in this region, with only 16 cases of septal lipoma reported in the literature as to our knowledge to date.

Histologically, lipomas consist of mature adipocytes that are enclosed in a thin fibrous capsule. According to WHO, it is classified into classic lipoma, fibrolipoma, angiolipoma, myxoid lipomas, spindle cell lipomas, pleomorphic lipomas, myelolipomas, lipomatosis of nerve, and lipoblastoma [3,6]. Forementioned variants are even more rare compared to the classic lipoma, with only four cases reported till now in nasal septal fibrolipoma [3,6,7]. Lipomas might be associated with other disorders like multiple hereditary lipomatosis, Gardner syndrome, adiposis dolorosa, and Madelung disease [2]. However, this association is very rare. It is thought that two-thirds of lipoma patients have some genetic abnormalities; the HMGA2 gene (located on 12q14.3) was linked to lipoma formation as well as specific structural rearrangements in chromosomes [2].

In the paediatric age group, lipomas are very rare, and if found, clinicians should rule out other associated intracranial lipomas. Pai syndrome is one of the rare syndromes that is associated with congenital lipoma, and it is characterized by the presence of cleft lip, lipomas, skin polyps, midline lipomas of the central nervous system, and other abnormal brain developments [1,3,5]. The aetiology of this syndrome still remains unclear till now, but some authors suggested autosomal dominant inheritance [2]. In our case, MRI showed no other associated pathologies intracranially.

Lipomas of the nasal septum can be asymptomatic or they can present with nasal symptoms or extra-nasal symptoms. Symptoms can include unilateral nasal obstruction, rhinorrhoea, reduction or complete loss of sense of smell, or epistaxis. Extra nasal symptoms may include facial paraesthesia or pain, headache, facial swelling, or palatal swelling [1,7].

The differential diagnosis of a unilateral nasal mass in general includes osteoma, haemangiomas, papilloma, angiofibroma, and other benign and malignant lesions. Imaging is important to rule out other differential diagnoses and to determine the exact size, location, extent of the mass, erosion or compression of the surrounding tissue, and other possible associated pathologies. In CT scans, lipoma shows a low-density lesion with peripheral enhancement. However, lipomas in MRI demonstrated high intensity lesions in T1 and T2 that are identical to subcutaneous fat with no enhancement and complete loss of signal with fat suppression [4,7].

Takasaki et al. reported in 2000 the first case of septal lipoma in an adult, and excision was done under local anesthesia with no signs of recurrence during the follow-up period [4]. Before that date, all septal lipoma cases were in infants and children [8,9]. There is one case reported where pre-operation biopsy was taken and it was reported as an inflammatory polyp, but after complete surgical resection, it came as a fibrolipoma of the posterior nasal septum [7].

The treatment of nasal septal lipoma is complete surgical excision. Some of the reported cases showed that the lipoma was adherent to the nasal mucosa and could not be separated from the mucosa [3]; on the other hand, there is one case in which the nasal mucosa was separable from the lipoma, and it was preserved [6]. In our case, the lipoma was adherent to the mucosa, and it was excised with the lipoma. If that is the case, reconstruction should be done depending on the extent and the structures involved.

Lipomas very rarely recur, and the rate of recurrence is less than 5% [6], so follow-up after resection is recommended. Lipomas are almost always benign lesions and transformation to liposarcoma is extremely rare [3,7,10], but the surgical resection is done mostly for aesthetic and functional reasons [10].

## Conclusions

Despite nasal septal lipoma being regarded as a very rare entity, it should be considered in patients with benign-looking unilateral nasal masses. Surgeons should keep in mind other syndromes associated with lipoma, like Pai syndrome or other intracranial pathologies. This is a unique case, as it showed challenges intraoperatively with adherent mass to the nasal mucosa and weak septal cartilage, which required proper reconstruction to prevent future nasal deformities.

## References

[1] Jahandideh H, Dehghani Firouzabadi F, Dehghani Firouzabadi M, Jan D, Roomiani M (2020). Lipoma of the nasal septum: a case report. Clin Case Rep.

[2] AbdollahiFakhim S, Bayazian G, Notash R (2014). Nasal septal lipoma in a child: Pai syndrome or not?. Int J Pediatr Otorhinolaryngol.

[3] Ozturk M, Ila K, Kara A, Iseri M (2013). Fibrolipoma of the nasal septum; report of the first case. J Otolaryngol Head Neck Surg.

[4] Takasaki K, Yano H, Hayashi T, Kobayashi T (2000). Nasal lipoma. J Laryngol Otol.

[5] Patil SB, Harsh S (2017). Lipoma of Columella with septal extension in Pai syndrome: report of a rare case. BMC Ear Nose Throat Disord.

[6] Jeong YW, Kim JY, Kim DY (2020). A case of fibrolipoma of the nasal septum. Korean J Otolaryngol-Head Neck Surg.

[7] Hollis LJ, Bailey CM, Albert DM, Hosni A (1996). Nasal lipomas presenting as part of a syndromic diagnosis. J Laryngol Otol.

[8] Aini NR, Mohamad S, Abdullah B (2021). Fibrolipoma presenting as a posterior nasal septum mass: a report of a unique case and review of the literature. Proceed Singapore Health.

[9] Morgan DW, Evans JN (1990). Developmental nasal anomalies. J Laryngol Otol.

[10] Funai MN, Risola CF, Gomes LM (2015). Fibrolipoma of the nasal septum: an unusual pediatric case. Int J Pediatr Otorhinolaryngol Extra.

